# External assessment of the EUROMACS right-sided heart failure risk score

**DOI:** 10.1038/s41598-021-94792-3

**Published:** 2021-08-09

**Authors:** Hirak Shah, Thomas Murray, Jessica Schultz, Ranjit John, Cindy M. Martin, Thenappan Thenappan, Rebecca Cogswell

**Affiliations:** 1 Department of Medicine, Division of Cardiology, University of Minnesota, 401 East River Parkway, Variety Club Research Center (VCRC), 1st Floor – Suite 131, Minneapolis, MN 55455 USA; 2 Division of Biostatistics, University of Minnesota, Minneapolis, MN USA; 3 Department of Cardiothoracic Surgery, Division of Surgery, University of Minnesota, Minneapolis, MN USA

**Keywords:** Cardiology, Cardiovascular diseases, Cardiomyopathies, Heart failure

## Abstract

The EUROMACS Right-Sided Heart Failure Risk Score was developed to predict right ventricular failure (RVF) after left ventricular assist device (LVAD) placement. The predictive ability of the EUROMACS score has not been tested in other cohorts. We performed a single center analysis of a continuous-flow (CF) LVAD cohort (n = 254) where we calculated EUROMACS risk scores and assessed for right ventricular heart failure after LVAD implantation. Thirty-nine percent of patients (100/254) had post-operative RVF, of which 9% (23/254) required prolonged inotropic support and 5% (12/254) required RVAD placement. For patients who developed RVF after LVAD implantation, there was a 45% increase in the hazards of death on LVAD support (HR 1.45, 95% CI 0.98–2.2, *p* = 0.066). Two variables in the EUROMACS score (Hemoglobin and Right Atrial Pressure to Pulmonary Capillary Wedge Pressure ratio) were not predictive of RVF in our cohort. Overall, the EUROMACS score had poor external discrimination in our cohort with area under the curve of 58% (95% CI 52–66%). Further work is necessary to enhance our ability to predict RVF after LVAD implantation.

## Introduction

Right ventricular (RV) failure remains common after left ventricular assist device placement (LVAD) even in contemporary continuous flow era^1^, and remains a leading cause of morbidity and mortality after LVAD placement^2,3^. A number of definitions of early right ventricular failure after LVAD exist. All include unplanned right ventricular assist device (RVAD) placement after LVAD implantation, however definitions vary by length and use of pulmonary vasodilators or intravenous inotropes^1,4,5^. Several prediction tools have been developed to try to capture risk of post-operative RV failure with pre-operative variables, however the performance of these models has been variable to poor on external validation^3,6^. One of the more recently published risk models was developed from the EUROMACS database^7^. This analysis included 2,988 patients implanted with LVADs in Europe in which 433 patients (21.7%) developed right ventricular failure. After performing logistic regression, a combination of five variables were found to be highly predictive of right sided heart failure. These variables include right atrial/pulmonary capillary wedge pressure > 0.54, hemoglobin < 10 g/dL, use of multiple intravenous inotropes prior to LVAD implantation, INTERMACS Class 1–3, and severe right ventricular dysfunction on echocardiography. The C-Statistic for this risk score was 0.7, which was higher than other risk scores that have been published. The performance of this score has been less than the development cohort in several small to intermediate sized external validation datasets (n = 93–194)^8–10^. The purpose of this study was to assess the performance of the EUROMACs score in a large, external continuous flow LVAD dataset and assess for other univariate predictors of RV failure.

## Methods

### Cohort and inclusion criteria

The institutional review board of the University of Minnesota Medical Center approved this study. The requirement of informed consent for the study is waived by the institutional review board of the University of Minnesota. All methods were carried out in accordance with relevant guidelines and regulations. At the time of this analysis, the larger continuous-flow LVAD cohort consisted of 451 patients implanted between 2007 and 2017. Of these, 254 patients met the following inclusion criteria 1) first time, continuous-flow LVAD implantation 2) complete pre-operative variables to complete EUORMACS score calculation, and 3) complete post-operative data to determine date of inotrope wean, pulmonary vasodilator wean and RVAD use.

The following demographic and clinical covariate data are available in the University of Minnesota LVAD database, which is updated through data extraction and manual chart review: age, gender, body mass index (BMI), serum creatinine, albumin, Interagency Registry for Mechanical Circulatory Support (INTERMACS) profile, pre-operative hemodynamics, bridge to transplant status, cardiomyopathy type, presence of diabetes, and NT pro b-type natriuretic peptide (NT-proBNP). Vital status was obtained from chart review. The date of the last clinic visit was recorded for patients who were still alive at the end of follow up. For cardiac transplantation, the date of cardiac transplant was obtained from the electronic medical record and confirmed with an operative report.

### Primary predictor and outcome

The primary predictor for this analysis was follow up EUROMACS RV score, which was calculated by totaling points for the following pre-LVAD clinical variables. RA/PCWP > 0.54: 2 points, hemoglobin ≤ 10 g/dL:1 point, multiple inotropes: 2.5 points, INTERMACS 1–3: 2 points, and severe RV dysfunction: 2 points. The primary outcome was early severe post-operative right ventricular failure defined as need for short/long-term mechanical right-sided circulatory support within 30 days of LVAD implantation, continuous inotropic support greater than or equal to 14 days, or need for pulmonary vasodilators for greater than 48 h.

### Statistical methods

All statistical analyses were performed using STATA 16 (College Station, Texas). A *p* value of < 0.05 was considered statistically significant. Baseline characteristics between cohort subjects who developed early RVF and those who did not develop early RVF were compared with normally distributed continuous variables were compared with t-tests and Wilcoxon rank sum tests for non-normally distributed continuous variables. Categorical variables were compared with Pearson chi-squared tests or Fishers exact test, where appropriate. In order to determine the relationship between baseline co-variates and follow up pulmonary capillary wedge pressure, multivariable linear regression analyses were performed.

In order to assess EUROMACS model discrimination, a receiver operator curve was generated with EUROMACS score as the predictor and post LVAD RVF as the outcome. This was repeated using the RVF definition of RVAD or prolonged inotropes use only. The EUROMACS score was also assessed as a predictor of RVF using logistic regression. In order to determine the relationship between other pre-operative variables and RVF, logistic regression was performed. The relationship between RVF (or RVAD use alone) and subsequent LVAD mortality was assessed with cox regression.

## Results

The mean age of the cohort was 59 years of age. Eighty two percent were male, 80% were Caucasian, and 51% were designated as bridge to transplantation. The majority of patients (52%) were INTERMACS profile 2–3. A total of 100 patients (39%) were diagnosed with early right ventricular failure and 154 patients (61%) did not have post-operative right heart failure. There were no significant differences in age, sex, race, baseline atrial fibrillation, cardiomyopathy type, diabetes, chronic obstructive pulmonary disease, LVAD surgical strategy, INTERMACS profile, pulmonary artery systolic pressure or cardiac index between both groups (Table 1). The 60 day mortality of the cohort was 10.6% (27/254). Of those, 15/27 (56%) were deaths attributable to right heart failure.

**Table 1:** Baseline characteristics of the cohort by presence or absence of early right heart failure by the.

Variable	Total cohort	No early RHF (N = 154)	Early RHF (N = 100)	*p* value
Age at LVAD implantation	62 (52–69)	62 (54–69) [0]	62 (50–68) [0]	0.35
Male	208 (82%)	127 (83%) [0]	81 (81%) [0]	0.87
Race White	204 (86%)	121 (85%)	83 (88%)	0.56
Race Black	20(8)	12 (9%)	8 (8.5%)	
Race other	12(5)	9 (6%)	3 (3.2%)	
Race missing	18	12	6	
Baseline atrial fibrillation	115(46%)	68 (45%) [2]	47 (48%) [1]	0.70
Ischemic cardiomyopathy	134 (53%)	78 (51%) [0]	56 (56%) [0]	0.44
Diabetes	122 (47%)	75 (49%) [2]	47 (48%) [1]	0.80
Chronic obstructive pulmonary disease	60 (24%)	35 (23%) [2]	25 (25%) [1]	0.76
Bridge to transplant	129 (51%)	79 (51.3%) [0]	50 (50%) [0]	0.90
INTERMACS 1	30 (12%)	14 (9%)	16 (16%)	0.14
INTERMACS 2–3	131 (52%)	78 (51%)	53 (53%)	
INTERMACS 4–7	93 (37%)	62 (40%)	31 (31%)	
Body Mass Index (kg/m^2^)	28.3 (25.2–33.0)	28 (25.0–32.4) [6]	29.3 (26.1–33.3) [6]	0.48
Creatinine (mg/dL)	1.2 (0.96–1.7)	1.1 (0.9–1.6) [2]	1.3 (1.1–1.8) [1]	0.015
Albumin (g/dL)	3.3 (2.5–3.7)	3.4 (3–3.8) [2]	3.3 (2.9–3.5) [1]	0.21
Right atrial pressure (mmHg)	11.5 (7–17)	10 (7–15) [19]	14 (10–18) [19]	0.03
Pulmonary artery systolic pressure (mmHg)	50 (40–60)	50 (40–60) [19]	50 (43–62) [19]	0.56
Pulmonary capillary wedge pressure (mmHg)	22 (17–28)	22(16–29) [19]	23 (18–28) [19]	0.06
Pulmonary artery diastolic pressure(mmHg)	25 (19–30)	23 (18–30) [19]	26 (21–32) [19]	0.15
Cardiac Index (L/min/m^2^)	1.9 (1.6–2.3)	1.9 (1.6–2.3) [14]	1.9 (1.5–2.3) [15]	0.97
Pulmonary arterial compliance (mL∙mmHg^−1^)	1.9 (1.4–2.7)	1.9 (1.4–2.9) [23]	1.8 (1.3–2.4) [21]	0.18
Pulmonary artery pressure index	2.2 (1.4–3.6)	2.4 (1.6–4) [19]	1.9 (1.3–3.1) [19]	0.02
Right ventricular stroke work index (g/m/beat/m^2^)	13.7 (10–17)	13.9 (11–17) [23]	13.4 (10–18) [21]	0.65
Severe right ventricular dysfunction by echo	40 (17)	18 (12%) [13]	22 (22%) [10]	0.18

Continuous variables are described with median (IQR) [N missing]. Binary variables are described with N (%) [N missing].

### Outcomes of right heart failure

**Figure 1:**
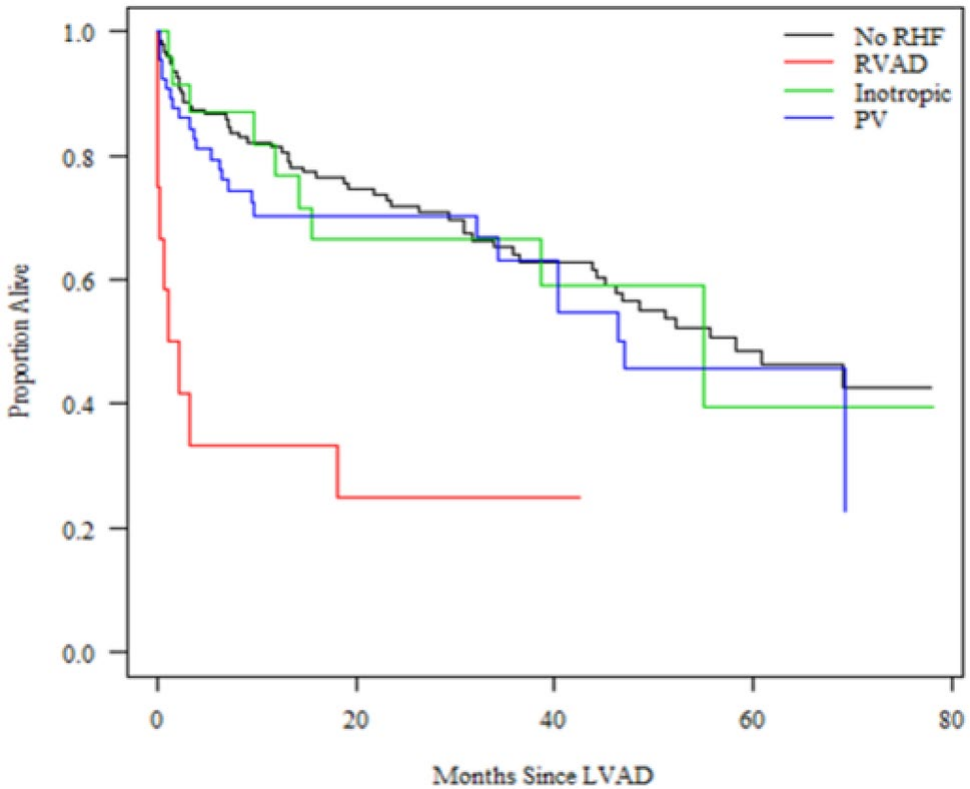
Kaplan Meier survival curves of LVAD recipients by type of right heart failure. RHF: right ventricular heart failure, RVAD: right ventricular assist device within 30 days, Inotropic: Inotrope Use > 14 days after LVAD, PV: pulmonary vasodilator Use > 48 h.

Of the 100 patients who met RV failure criteria, 12 patients required RVAD and 23 patients required inotropes > 14 days. Patients who required pulmonary vasodilators > 48 h comprised the vast majority (n = 65) of patients who were defined as having post-operative RVF. Early right-sided heart failure was associated with increased mortality (unadjusted HR 1.45; 95% CI 0.98–2.16, *p* = 0.066). Use of RVAD after LVAD surgery was the primary driver of increased mortality (HR 4.56; 95% CI 2.28–9.11, *p* < 0.001, Fig. 1).

### Risk factor for early postoperative right heart failure

We tested 66 distinct risk factors for early post-operative right heart failure. An additional nine factors were calculated from these 66 variables (Tables 2, 3, 4). Of the individual variables tested, a higher pulmonary arterial pressure was protective against RV failure as was a higher pulmonary arterial pulse pressure. Higher creatinine, lower albumin, and higher total bilirubin were all associated with increased risk of RV failure after LVAD. Pre-operative ventilator support, multiple inotropes, extra corporal membrane oxygenator support, and non-elective IABP were also associated with higher risk of RVF. Of note, hemodynamic parameters such as right atrial pressure, right atrial/pulmonary capillary wedge pressure, pulmonary artery pulsatility index, pulmonary artery compliance, and pulmonary artery elastance were not associated with increased risk of right sided heart failure in this dataset.

**Table 2:** Odds-ratio of each standardized continuous risk factor for early right heart failure based on univariate logistic regression models.

Predictor	Odds-ratio (95% CI)	*p* value
Age (years)	0.53 (0.19, 1.47)	0.221
Body Mass Index (kg/m^2^)	1.19 (0.38, 3.73)	0.761
log NT-proBNP (pg/mL)	1.06 (0.36, 3.12)	0.910
Glomerular filtration rate (mL/min)	0.18 (0.05, 0.66)	0.010*
Hemoglobin (g/dL)	0.51 (0.15, 1.81)	0.300
International normalized ratio	2.77 (1.28, 6.00)	0.010*
Platelets	0.79 (0.24, 2.66)	0.707
Creatinine (mg/dL)	3.71 (1.63, 8.46)	0.002*
C-reactive protein	2.49 (1.09, 5.72)	0.031*
Prealbumin (mg/L)	0.21 (0.05, 0.85)	0.029*
Sodium	1.33 (0.41, 4.30)	0.638
Albumin(mg/dL)	0.22 (0.07, 0.69)	0.010*
ALT	1.80 (1.02, 3.17)	0.041*
AST	2.08 (0.94, 4.58)	0.070
Total bilirubin (mg/d)	1.93 (1.03, 3.59)	0.039*
Total cholesterol (mg/dL)	3.46 (1.27, 9.40)	0.015*
High density lipoprotein (mg/dL)	1.01 (0.32, 3.23)	0.981
White blood cell count	2.31 (1.01, 5.28)	0.047*
Partial thromboplastin time	1.65 (0.73, 3.75)	0.228
log NT-proBNP (pg/mL)	1.06 (0.36, 3.12)	0.910
Heart rate (beats/min)	1.22 (0.39, 3.82)	0.736
Diastolic blood pressure (mmHg)	0.73 (0.25, 2.19)	0.578
Ejection fraction (%)	2.10 (0.74, 5.94)	0.161
Left ventricular end diastolic dimension (cm)	0.85 (0.27, 2.69)	0.776
Left ventricular end systolic dimension (cm)	0.62 (0.19, 1.97)	0.417
International normalized ratio	2.77 (1.28, 6.00)	0.010*
Mean arterial pressure (mmHg)	0.55 (0.20, 1.54)	0.256
Systolic blood pressure (mmHg)	0.51 (0.18, 1.47)	0.215
Cardiac output (L/min)	1.34 (0.43, 4.13)	0.615
Right atrial pressure (mmHg)	1.04 (0.33, 3.31)	0.949
Pulmonary artery systolic pressure (mmHg)	0.21 (0.06, 0.77)	0.019*
Pulmonary capillary wedge pressure (mmHg)	0.40 (0.12, 1.34)	0.140
Pulmonary artery diastolic pressure (mmHg)	0.48 (0.14, 1.63)	0.239
Pulmonary pulse pressure (mmHg)	0.22 (0.06, 0.76)	0.016*
Pulmonary artery elastance (mmHg/mL)	0.49 (0.12, 2.04)	0.327
Pulmonary artery compliance (mL∙mmHg^−1^)	1.66 (0.77, 3.55)	0.193
Pulmonary artery pulsatility index	0.21 (0.02, 2.53)	0.218
Right atrial: wedge pressure ratio	1.51 (0.56, 4.07)	0.415
Right ventricular end diastolic pressure (mmHg)	1.76 (0.57, 5.48)	0.327
Systolic blood pressure (mmHg)	0.51 (0.18, 1.47)	0.215
Stroke volume (mL)	1.10 (0.36, 3.31)	0.870
EUROMACS score	6.88 (2.31, 20.45)	0.001*

Each odds ratio estimate reflects a 2 standard deviation change in the corresponding variable.

**Table 3:** Odds-ratios of each binary risk factor for early right heart failure based on univariate logistic regression models.

Predictor	Odds-ratio (95% CI)	*p* value
Sex	2.51 (0.32, 19.96)	0.384
Bridge to transplant LVAD	0.68 (0.21, 2.2)	0.520
Diabetes mellitus	0.51 (0.15, 1.73)	0.280
Non-ischemic cardiomyopathy type	0.28 (0.07, 1.07)	0.063
Coronary artery disease	0.39 (0.12, 1.29)	0.123
Atrial fibrillation	0.83 (0.26, 2.68)	0.755
History of coronary artery bypass surgery	0.77 (0.16, 3.62)	0.739
Chronic obstructive pulmonary disease	0.62 (0.13, 2.91)	0.545
Hypercholesterolemia	1.14 (0.36, 3.64)	0.823
Hypertension	0.85 (0.27, 2.7)	0.779
Implanted cardiac defibrillator	0.55 (0.16, 1.89)	0.340
History of smoking	0.85 (0.18, 4.02)	0.840
Ventilator use	4.34 (1.22, 15.45)	0.023*
Continuous renal replacement therapy	0 (0, Inf)	0.992
Inotropes	2.76 (0.73, 10.45)	0.135
Multiple inotropes	10.52 (3.12, 35.53)	< 0.001*
Extra corporal membrane oxygenation	5.87 (1.42, 24.32)	0.015*
Impella	10.91 (0.92, 129.67)	0.058
Intra-aortic balloon pump (non-elective)	3.25 (1.01, 10.46)	0.049*

**Table 4:** Likelihood ratio test *p* value corresponding to each categorical risk factor for early right heart based on univariate logistic regression models (omitting odd-ratios as there are multiple per variable).

Predictor	Odds-ratio (95% CI)	*p* value
Race	–	0.523
INTERMACS profile	–	0.142
Right ventricle function	–	0.307
Right ventricle size	–	0.890
Tricuspid regurgitation grade	–	0.293

INTERMACS: interagency registry for mechanical circulatory support.

### Validation of the EUROMACS score

**Figure 2:**
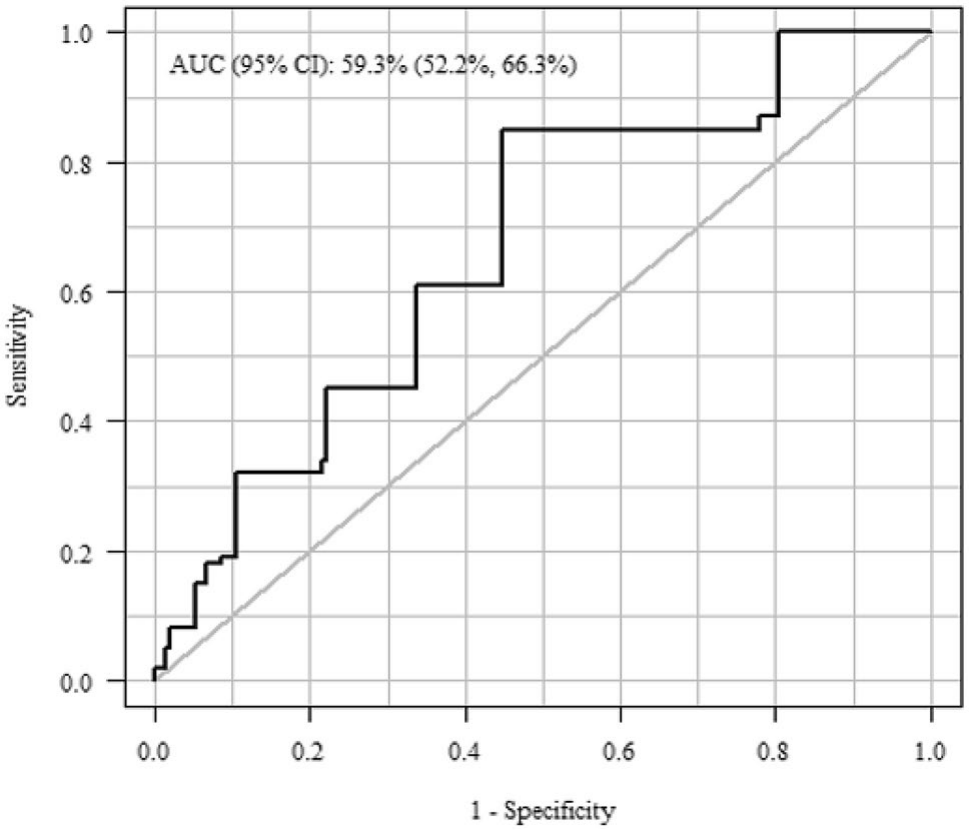
Receiver operator curve for the performance of the EUROMACS right ventricular risk score in an external, validation cohort. AUC: area under the curve, with 95% confidence interval.

An elevated EUROMACS score was associated with increased risk of RVF (OR 6.88; 95% CI 2.31 to 20.45, *p* = 0.001). The EUROMACS score had an area under the curve of 59%, (95% CI 52–66%) (Fig. 2). The performance of the EUROMACS score to predict RVF as defined by RVAD use or prolonged inotrope use was 67% (95% CI 54–79).

## Discussion

In this external validation of the EUROMACs score to predict right ventricular failure after CF-LVAD, we found we found that the EUROMACS score had relatively poor discrimination in predicting RV failure. Right ventricular failure was significantly associated with mortality after CF-LVAD in this dataset, which was mainly driven by the need for RVAD and/or prolonged inotropes. Many of the variables used in the EUROMACs score were not associated with RV failure in our cohort including hemoglobin and RA/PCWP ratio.

Right heart failure is one of the most common causes of early morbidity and mortality after CF-LVAD implantation. Predicting RV failure is important as planned institution of RVAD during LVAD surgery has been associated with improved outcomes compared to delayed RVAD implantation^11,12^, and a direct to transplant strategy can be employed in eligible patients to avoid this severe complication. The present study again demonstrates the difficulty in predicting RV failure after LVAD, even with the most contemporary risk scores. Our results mirror external validations of the EUROMACS right sided heart failure risk score where ROCs were in the 0.65 range^8–10^.

There are many reasons why our results may have differed from the original EUROMACs analysis. In our cohort, the incidence of the RV failure occurred in 39% patients undergoing CF-LVAD implantation. The majority of the patients (65%) met this definition due to prolonged pulmonary vasodilator use, while the EUROMACs data only had 1% of patients with prolonged pulmonary vasodilator use. RV failure defined by prolonged vasodilator use was not associated with increased mortality in our cohort, which questions the clinical significance of this part of the RV failure definition. The use of pulmonary vasodilators and time course of weaning these medications appears to be different between the University of Minnesota and EUROMACS derivation cohorts, which reflects significant variability in practice. When restricting the RVF definition to inotropes ≥ 14 days and/or need for RVAD, the performance of the EUROMACS score improved. Another difference between the cohorts was the lower numbers of destination therapy CF-LVAD implantations in the EUROMACs cohort (14% vs. 49%). The larger number of patients with comorbid conditions in the University of Minnesota dataset may have explained some of the lower performance observed in the model, as these populations have real differences.

There have been several previous studies designed to predict those patients at high likelihood of developing RV failure following LVAD implantation. Many of these include hemodynamic variables such as right atrial pressure, pulmonary artery pulsatility index, and right atrial pressure: pulmonary capillary wedge pressure ratio^13,14^. Other studies have shown that certain echocardiographic features of RV dysfunction such as TAPSE and semi-quantitative RV function are predictive of RV failure after LVAD^15,16^. In our cohort, none of the previously mentioned hemodynamic variables were predictive of RV failure. Similar to previous analyses, higher creatinine, lower albumin, and higher total bilirubin were all associated with increased risk of RV failure after LVAD. Pre-operative ventilator support, multiple inotropes, extra corporal membrane oxygenation, and non-elective IABP were also associated with higher risk of RVF in this cohort. All of the associated variables are direct or indirect markers of patient acuity, and may suggest longer standing heart failure is a risk factor for post-operative RV failure.

There are many reasons why published risk scores may perform poorly in validation cohorts. First, RV failure after LVAD does not have a universal definition^17^. For example, the widely used INTERMACS criteria for RV failure does not include pulmonary vasodilators as definition for RVF. With regard to the hemodynamic variables, these can change dramatically over a 24 h period as patient are managed with diuretics, inotropes and temporary support. Lastly, insults that occur to the RV in the operating room, such as RV ischemia from hypotension, prolonged cardiopulmonary bypass time, surgical positioning of the inflow cannula, fluid resuscitation and blood transfusions are not predictable ahead of time^18^. These intraoperative conditions can “unmask” underlying RV failure that might not have been predicted with traditional risk scores using clinical, hemodynamic, and echocardiographic data.

This study has several limitations. First, this was a single-center observational analysis. Second, our sample size is smaller than the EUROMACs cohort, which could limit the power of our study to determine variables that are associated with severe RV failure.

## Conclusion

The EUROMACS Right-Sided Heart Failure Risk Score had poor external discrimination on external validation. In the present cohort, variables associated with long-standing heart failure (creatinine, bilirubin, albumin) were more predictive of right ventricular heart failure. As RV failure post LVAD is a complex syndrome influenced by pre-operative, intra-operative, and post-operative factors, it remains difficult to predict. Further work will be required to enhance our understanding of the post-LVAD right ventricular failure syndrome.

## Abbreviations

BNP: Brain natriuretic peptide
BMI: Body mass index
COPD: Chronic obstructive pulmonary disease
EUROMACS: European Registry for Patients with Mechanical Circulatory Support
INR: International normalizing ratio
INTERMACS: Interagency Registry for Mechanical Circulatory Support
LVAD: Left ventricular assist device
RVF: Right ventricular failure
